# Clinical, Biochemical, Molecular, and Outcome Features of Mitochondrial 3-Hydroxy-3-Methylglutaryl-CoA Synthase Deficiency in 10 Chinese Patients

**DOI:** 10.3389/fgene.2021.816779

**Published:** 2022-03-04

**Authors:** Shengnan Wu, Linghua Shen, Qiong Chen, Chunxiu Gong, Yanling Yang, Haiyan Wei, Bingyan Cao, Yongxing Chen

**Affiliations:** ^1^ Department of Endocrinology and Metabolism, Henan Key Laboratory of Children’s Genetics and Metabolic Diseases, Children’s Hospital Affiliated to Zhengzhou University, Henan Children’s Hospital, Zhengzhou Children’s Hospital, Zhengzhou, China; ^2^ Department of Endocrinology, Genetics and Metabolism, Beijing Children’s Hospital, Capital Medical University, National Center for Children’s Health, Beijing, China; ^3^ Department of Pediatrics, Peking University First Hospital, Beijing, China

**Keywords:** 3-hydroxy-3-methylglutaryl-CoA synthase deficiency, hypoglycemia, ketogenesis, HMGCS2 gene, HMGCS2D

## Abstract

**Background:** Mitochondrial 3-hydroxy-3-methylglutaryl-CoA synthase deficiency (HMGCS2D) is a rare autosomal recessive metabolic disorder caused by mutations of the *HMGCS2* gene. To date, no more than 60 patients have been reported throughout the world.

**Purpose**: To analyze the clinical, biochemical, molecular, and outcome features of HMGCS2D in a case series of 10 new Chinese patients.

**Methods:** This retrospective study includes 10 Chinese patients diagnosed with HMGCS2D. We collected and analyzed clinical data for all patients. We also reviewed clinical data for 39 cases that had been reported previously.

**Results:** All of our patients had experienced their first metabolic crisis before 12 months old. The most common clinical manifestations were anorexia, dyspnea, and disturbance of consciousness (10/10), followed by vomiting (8/10), fever (7/10), cough (4/10), diarrhea, and seizures (3/10). Each patient (10/10) had a different degree of hepatomegaly and increased aminotransferase, severe metabolic acidosis, and hypofibrinogenemia. 9 patients presented with severe hypoglycemia and weak positives on qualitative tests of urinary ketone body. Patient 3 was the only one without hypoglycemia. Five patients had hypocalcemia, five patients had hyperammonemia, four patients had hyperuricemia, and three had hypertriglyceridemia. During the metabolic acidosis episode, we observed high dicarboxylic acid values in urine, and the elevated ratio of blood acetylcarnitine to free carnitine may have been an additional biochemical signature. However, all returned to normal during the interictal interval. Molecular analysis identified 15 variants in the *HMGCS2* gene, of which 10 were novel (c.220G>A/p.E74K, c.407A>G/p.D136G, c.422T>A/p.V141D, c.719A>C/p.D240A, c.821G>A/p.R274H, c.39dupA/p.L14Tfs*59, c.1394delA/p.N465Tfs*10, c.788delT/p.L263Cfs*36, c.717T>G/p.Y239*, and c.1017-2A>G). Combining these with previous cases, the known mutation c.1201G>T/p.E401* has been found in 6/40 (15.0%) of mutated alleles in 21 Chinese patients from 20 families, while none have been found in other populations. We found that patients with biallelic truncation mutation appeared to show a more severe clinical condition through a literature review.

**Conclusion:** This study analyzed the phenotypic and genetic features of HMGCS2D in a Chinese case series. We also expanded the *HMGCS2* mutational spectrum with 10 novel variants. The c.1201G>T/p.E401* mutation was the most frequent, representing 15.0% of the mutated alleles in reported unrelated Chinese patients, and thus, it may be a hot spot mutation.

## Introduction

Mitochondrial 3-hydroxy-3-methylglutaryl-CoA synthase deficiency (mitochondrial HMG-CoA synthase deficiency; HMGCS2D; OMIM#605911) is a rare autosomal recessive metabolic disorder caused by mutations in the *HMGCS2* gene (OMIM*600234). The *HMGCS2* gene encodes mitochondrial HMG-CoA synthase, which catalyzes the reactions of acetyl-CoA and acetoacetyl-CoA into HMG-CoA during ketone body synthesis. Ketone bodies are an important energy supply for the brain, heart and kidneys during long-term fasting and carbohydrate deprivation. Pathogenic mutations on the *HMGCS2* gene cause mitochondrial HMG-CoA synthase deficiency which disrupts the ketogenesis and blocks the energy supply to the brain during the fasting state. Patients present with intermittent vomiting, lethargy, respiratory distress, encephalopathy, and hepatomegaly, usually precipitated by an intercurrent infection or prolonged fasting (Aledo et al., 2006). Hypoketotic hypoglycemia, metabolic acidosis, increased transaminitis and dicarboxylic aciduria are common laboratory findings during the acute attack stage. Nevertheless, the clinical manifestations and biochemical abnormalities usually return to normal during the interictal period. In 1997, Thompson described the first case of mitochondrial HMGCS2D (Thompson et al., 1997). To date, no more than 60 patients carrying over 40 variants have been reported throughout the world among different ethnic groups. Among them, only 11 patients, from 10 families, are of Chinese descent.

In the present study, we report 10 new Chinese patients with HMGCS2D, and summarize their clinical, biochemical, molecular and outcome characteristics. We reviewed 39 patients with relatively complete clinical data reported for overall understanding of the molecular genetics features of Chinese patients, and to explore the correlation between genotype and clinical features and disease severity. This has not been reported in previous studies.

## Materials and Methods

### Subjects and Clinical Evaluation

Ten unrelated patients (two boys and eight girls) from different Chinese families identified between 2015 and 2021 were included in this retrospective analysis. They had each been diagnosed with HMGCS2D based on clinical features (encephalopathy, and hepatomegaly, usually precipitated by an intercurrent infection or prolonged fasting), biochemical detection results (hypoketotic hypoglycemia, transaminitis, metabolic acidosis and dicarboxylic aciduria), as well as molecular analysis. We collected clinical data through the review of patients’ medical records, including the age at onset, primary clinical symptoms, medical management, and biochemical and clinical outcomes following therapy.

### Biochemical Analysis

We performed routine blood and urine examination to assess blood gas analysis, ammonia, glucose, liver function, renal function, blood lipids and plasma electrolytes. Then, we applied tandem mass spectrometry to test serum amino acids and acylcarnitines, and analyzed the results using ChemoView software. To analyze urine organic acids, we used gas chromatography-mass spectrometry and the Inborn Errors of Metabolism Screening System. We also performed abdominal ultrasonography and cranial MRI on most patients.

### Molecular Analysis

We extracted genomic DNA from peripheral blood leucocytes using a QIAamp DNA Blood Midi kit (Qiagen, Hilden, Germany), and generated sequences using the Agilent Bioanalyzer. Then, we applied whole-exome sequencing for mutation screening. Additionally, we performed Sanger sequencing validation on all patients who we found to be harbouring gene mutations, as well as their parents. The pathogenicity of novel variants was evaluated according to the American College of Medical Genetics and Genomics (ACMG) standards and guidelines (Richards et al., 2015). For the novel mis-sense variants, multiple sequence alignment studies were performed to verify the amino acid conservation using the UCSC Genome Bioinformatics Database, and potential pathogenicity was analyzed using Mutation taster, PolyPhen-2, and Sorting Intolerant From Tolerant (SIFT). Swiss model (https://swissmodel.expasy.org/) was also used to predict the protein 3D structure and evaluate the impact of these novel missense variants on protein structure.

### Treatment, Follow Up and Outcome Evaluation

When initial metabolic decompensation occurred, all patients were admitted to the hospital and received regular supportive treatment including intravenous glucose and sodium bicarbonate. We also administered liver-protecting and carnitine therapy. We gave mechanical ventilation and continuous renal replacement therapy to any patients who were critically ill because of persistent metabolic acidosis and multiple organ dysfunction. Once the HMGCS2D diagnosis was confirmed, all patients were instructed to avoid long periods of fasting, and put on a low-fat diet.

All patients were enrolled into a simple follow-up study. During the follow-up period, each patient had several telephone survey or outpatient visits. We performed physical growth and psychomotor evaluation, as well as routine laboratory tests such as urine organic acids, plasma amino acids and acylcarnitines and other biochemical investigations, every 6–12 months. Psychomotor evaluation was based on the intellectual test results according to Gesell Developmental Schedules at the last follow-up.

### Case Review and Grouping

For overall understanding of Chinese patients’ molecular genetics features, and to explore the correlation between genotype and clinical features and disease severity, we reviewed 39 previously reported patients with relatively complete clinical data, including the genetic analysis results. In our study, we divided the patients into 3 phenotypic groups according to their clinical severity: A deceased group, a severe group, and a mild group. The deceased group included patients who had died because of disease onset. The severe group included patients who met one of the following conditions: Recurrent attacks on multiple occasions; blood pH value lower than 7.0 in metabolic decompensation; had required mechanical ventilation or CRRT treatment in metabolic decompensation; had become cognitively disabled after disease onset. The mild group included patients with milder symptoms. According to their disease-causing variation type, we divided them into 3 genotypic groups: The A group included patients carrying 2 truncating mutations on the *HMGCS2* gene; the B group included patients carrying only 1 truncating mutation on the *HMGCS2* gene; and the C group included patients carrying no truncating mutations.

### Statistical Analysis

We performed statistical analysis in SPSS 22.0. We analyzed the correlations between different phenotypic groups and genotypic groups with a Cochran-Mantel-Haenszel test. We considered any results with *p* < 0.05 as having a statistically significant difference.

## Results

### Patients’ Clinical Characteristics, Biochemical Detection

As summarized in Table 1, there were 8 females and 2 males in our study. All of their parents were non-consanguineous. Among the 10 cases, patient 1 had a deceased sister who had died of a sudden coma after respiratory infections at the age of 8 months, while patient 4 had an older brother who had died of Reye Syndrome at 1 year and 10 months. For patient 10, his mother had experienced spontaneous abortion in her first two pregnancies with no specific cause. None of the other patients had positive family history. Patients had experienced their first metabolic crises at ages ranging from 5 to 12 months. The most common clinical manifestations were anorexia, fever and cough, which then developed into vomiting, dyspnea and disturbance of consciousness. The time from onset to progression to dyspnea and disturbance of consciousness ranged from 2 to 7 days. During the course of disease, all patients had anorexia, dyspnea and disturbance of consciousness (10/10). The second most common symptom was vomiting (8/10), followed by fever (7/10), cough (4/10), diarrhea and seizures (3/10).

**TABLE 1:** Clinical information, biochemical assays, and outcome findings of 10 new patients with HMGCS2D.

		Patient 1	Patient 2	Patient 3	Patient 4	Patient 5	Patient 6	Patient 7	Patient 8	Patient 9	Patient 10
Gender		Female	Female	Female	Female	Female	Female	Female	Male	Female	Male
Age at onset		11.5m	5m	9m	12m	12m	6m	9m	8m	10m	12m
Anorexia		+	+	+	+	+	+	+	+	+	+
Vomiting		+	−	−	+	+	+	+	+	+	+
Diarrhea		−	−	−	−	+	−	−	+	+	−
Fever		+	−	+	+	+	+	+	−	+	−
Cough		−	+	+	+	−	-	−	+	−	−
Dyspnea		+	+	+	+	+	+	+	+	+	+
Consciousness disorder		+	+	+	+	+	+	+	+	+	+
Seizure		−	−	−	+	−	+	−	−	+	−
Blood glucose (mmol/L)		2.3	2.4	9.3	0.6	2.0	0.3	1.8	1.2	0.12	0.3
Blood gas analysis		PH 7.03	PH 7.07	PH 6.79	PH 6.81	PH 7.30	PH 7.12	PH 7.03	PH 7.01	PH 6.9	PH 7.046
		BE -25	BE -25.5	BE -31	BE -28.5	BE -17	BE -23.4	BE -26	BE -31	BE -30	BE -27.5
		AG 35	AG 29	AG 48	AG 67	AG 37	AG 39	AG 38	AG 30.8	AG 35.8	AG 39.3
Lactic acid (mmol/L)		0.8	0.5	0.4	0.6	0.4	0.69	0.7	0.8	0.5	1.9
Blood ammonia (umol/L)		123	131.5	NA	468	48	8	NA	NA	166.3	NA
Hepatomegaly		+	+	+	+	+	+	+	+	+	+
ALT (U/L)		206	94	82	3,216	1,333	57	44	99	91	91
AST (U/L)		247	135	113	6,608	1893	120	113	167	128	105
Hyperuricemia		−	−	NA	+	−	−	−	+	+	+
Hypocalcemia		+	-	−	+	−	−	−	+	+	+
Hypofibrinogenemia		+	+	+	+	+	+	NA	NA	+	+
Triglyceride (mmol/L)		NA	5.37	NA	8.34	−	NA	−	2.69	NA	NA
CK (U/L)		154	198	106	239	492	133	157	171	304	105
CKMB (U/L)		22	62.3	43	34.6	192	31	19	39	44	67
C0 (free carnitine)		Low	Low	Low	Low	Low	Low	Low	Normal	Normal	Normal
C2 (acetylcarnitine)		Elevated	Elevated	Elevated	Elevated	Elevated	Normal	Elevated	Elevated	Elevated	Elevated
Dicarboxylic aciduria		+	+	+	+	+	+	+	+	+	+
Ketonuria		Mild	Mild	Mild	Mild	Mild	Mild	Mild	Mild	Mild	Mild
Brain MRI		Widening of sulcus and bilateral frontal and temporal subarachnoid space	Abnormal myelination of white matter	Abnormal signal in bilateral basal ganglia; broaden bilateral frontal and temporal subarachnoid space	NA	NA	Normal	NA	Normal	Normal	Abnormal signal in bilateral basal ganglia
Mechanical ventilation		+	+	+	+	−	−	+	+	+	+
CRRT		−	−	+	+	−	−	−	+	−	+
At last follow-up	Age	5y1m	5y7m	3y2m	—	3y	4y10m	6y10m	2y2m	2y	1y7m
	Height (cm)	114.8	111.3	97.1	—	98.5	107.5	120.9	88.5	85.2	85.8
	Weight (kg)	22.6	18.2	14.7	—	15.2	17.7	19.5	12.6	11	13
	DQ score	93	92	90	—	94	ND	ND	91	96	93
Variation (Allele 1)		▲c.39dupA/p.L14Tfs*59 (Exon 1)	▲c.422A>T/p.V141D (Exon 2)	▲c.821G>A/p.R274H (Exon 4)	▲c.1394delA/p.N465Tfs*10 (Exon 8)	c.160G>A/p.V54M (Exon 2)	c.1502G>A/p.R501Q (Exon 9)	c.1201G>T/p.E401* (Exon 7)	c.1201G>T/p.E401* (Exon 7)	c.1201G>T/p.E401* (Exon 7)	▲c.788delT/p.L263Cfs*36 (Exon 4)
Variation (Allele 2)		▲c.220G>A/p.E74K (Exon 2)	c.559+1G>T (Intron 2)	c.1187+1G>C (Intron 6)	c.1201G>T/p.E401* (Exon 7)	▲c.220G>A/p.E74K (Exon 2)	▲c.719A>C/p.D240A (Exon 4)	c.559+1G>T (Intron 2)	▲c.1017-2A>G (Intron 5)	▲c.407A>G/p.D136G (Exon 2)	▲c.717T>G/p.Y239* (Exon 4)

Abbreviations and reference range: ALT, alanine aminotransferase; AST, aspartate aminotransferase; CK, creatine kinase; CKMB, creatine kinase isoenzyme; CRRT, continuous renal replacement therapy; DQ, development quotient; NA, not available; ND, not detected; ▲ novel variation. Reference range: ALT: 0-40U/L; AST: 0-40U/L; CK: 20-200U/L; CKMB: 0-24U/L; Triglyceride: 0.38-2.25 mmol/L; Lactic acid: 0–2.2 mmol/L; Blood ammonia: 9–33umol/L.

Routine biochemical laboratory results at onset are shown in Table 1. Each patient had a different degree of hepatomegaly, about 2–7 cm below the right costal margin. Laboratory examinations showed severe metabolic acidosis (10/10), increased aminotransferase from mild to severe (10/10), hypofibrinogenemia (10/10), slightly increased creatine kinase isoenzyme (8/10), increased creatine kinase (3/10), and normal lactate (10/10). Among all patients, 9 presented with severe hypoglycemia and weak positives in qualitative urinary ketone body tests, while we observed no hypoglycemia in Patient 3. Five patients (Patient 1, 2, 4, 5 and 9) had hyperammonemia. Five (Patient 1, 2, 4, 9 and 10) had hypocalcemia. Three (Patient 2, 4, and 8) had hypertriglyceridemia. Eight received cranial MRI examinations, and 3 cases (Patient 1, 2 and 3) showed widening of the sulcus and bilateral frontal and temporal subarachnoid space, two (Patient 3 and 10) showed abnormal signal in the basal ganglia, one (Patient 2) showed delayed myelination in white matter, while four (Patient 6, 7, 8 and 9) were normal. During acute metabolic decompensation, eight patients (all except for Patient 5 and 6) received mechanical ventilation for respiratory failure resulting from serious metabolic acidosis. Four (Patient 3, 4, 8, and 10) had to undergo continuous renal replacement therapy (CRRT) because of serious and/or obstinate metabolic acidosis.

We performed urine organic acid analysis during the acute episode of metabolic acidosis. While we detected a massive amount of dicarboxylic acid in all patients, ketone body excretion was only mildly elevated. Plasma amino acids and acylcarnitine analysis revealed that free carnitine levels were low with a mildly increased acetylcarnitine in Patients 1–3, 5 and 7. Patients 4 and 6 had decreased free carnitine but normal acetylcarnitine, while Patients 8–10 had normal free carnitine but elevated acetylcarnitine.

### Molecular Genetic Analysis

We performed molecular analysis on all patients, and detected biallelic mutations in the *HMGCS2* gene in all of them. In our study, we identified fifteen molecular variants in the *HMGCS2* gene, ten of which were novel. Five were known mutations, including 2 missense mutations (c.160G > A/p.V54M, c.1502G > A/p.R501Q), 2 splicing mutations (c.559+1G > T, c.1187+1G > C) and one nonsense mutation (c.1201G > T/p.E401*). The ten novel potentially pathogenic variants have not been previously reported in the literature or registered in the HGMD, ClinVarMiner, ExAC or gnomAD databases. This includes the 5 missense mutations (c.220G > A/p.E74K, c.407A > G/p.D136G, c.422T > A/p.V141D, c.719A > C/p.D240A, c.821G > A/p.R274H), 3 frame-shift mutations (c.39dupA/p.L14Tfs*59, c.1394delA/p.N465Tfs*10, c.788delT/p. L263Cfs*36), one nonsense mutation (c.717T > G/p.Y239*) and one splicing mutation (c.1017-2A > G). Multiple sequence alignment studies suggested all the amino acids were conserved. Multiple lines of computational evidence supported a deleterious effect on the gene about the novel mutations. Detailed results of the 15 variants and prediction of effects of some novel mutations are shown in Table 2. In addition, we have performed a computational evaluation of the impact of the novel missense variants on protein structural using Swiss-model software. The results show that the 3D structure of HMGCS2 protein may be affected by these 5 novel mis-sense variants. Detailed results were shown in Supplementary Figure S1.

**TABLE 2:** Molecular analysis of *HMGCS2* gene identified in 10 new Chinese patients with HMGCS2D.

Case	Region	Nucleotide change	Amino-acid change	MutationTaster prediction	SIFT prediction (score)	Polyphen-2.0 prediction (score)	Conservation	Novel or reported	Assession number
Patient 1	Exon 1	c.39dupA	p.L14Tfs*59	—	—	—	Yes	Novel	SCV002032067
	Exon 2	c.220G>A	p.E74K	Disease causing	Damaging (0.008)	Probably damaging (0.999)	Yes	Novel	SCV002032068
Patient 2	Exon 2	c.422T>A	p.V141D	Disease causing	Damaging (0)	Probably damaging (1.00)	Yes	Novel	SCV002032069
	Intron 2	c.559+1G>T	—	Disease causing	—	—	Yes	Reported	rs587603096
Patient 3	Exon 4	c.821G>A	p.R274H	Disease causing	Tolerated (0.166)	Probably damaging (0.999)	Yes	Novel	SCV002032070
	Intron 6	c.1187+1G>C	—	Disease causing	—	—	Yes	Reported	rs764706394
Patient 4	Exon 8	c.1394delA	p.N465Tfs*10	—	—	—	Yes	Novel	SCV002032071
	Exon 7	c.1201G>T	p.E401*	Disease causing	—	—	Yes	Reported	rs1454719802
Patient 5	Exon 2	c.160G>A	p.V54M	Disease causing	Damaging (0)	Probably damaging (1.00)	Yes	Reported	rs28937320
	Exon 2	c.220G>A	p.E74K	Disease causing	Damaging (0.008)	Probably damaging (0.999)	Yes	Novel	SCV002032068
Patient 6	Exon 9	c.1502G>A	p.R501Q	Disease causing	Damaging (0)	Probably damaging (1.00)	Yes	Reported	rs372079931
	Exon 4	c.719A>C	p.D240A	Disease causing	Damaging (0)	Probably damaging (1.00)	Yes	Novel	SCV002032072
Patient 7	Exon 7	c.1201G>T	p.E401*	Disease causing	—	—	Yes	Reported	rs1454719802
	Intron 2	c.559+1G>T	—	Disease causing	—	—	Yes	Reported	rs587603096
Patient 8	Exon 7	c.1201G>T	p.E401*	Disease causing	—	—	Yes	Reported	rs1454719802
	Intron 5	c.1017-2A>G	—	Disease causing	—	—	Yes	Novel	SCV002032073
Patient 9	Exon 7	c.1201G>T	p.E401*	Disease causing	—	—	Yes	Reported	rs1454719802
	Exon 2	c.407A>G	p.D136G	Disease causing	Damaging (0)	Probably damaging (1.00)	Yes	Novel	SCV002032074
Patient 10	Exon 4	c.788delT	p.L263Cfs*36	—	—	—	Yes	Novel	SCV002032075
	Exon 4	c.717T>G	p.Y239*	Disease causing	—	—	Yes	Novel	SCV002032076

### Outcome Evaluation

Except for Patient 4 who died of multiple organ failure due to severe metabolic disorder at onset, all patients had complete recovery with symptomatic and supportive treatment. Each maintained well-being by avoiding long-term fasting and taking glucose supplements during illness. Hitherto, all 9 cases had followed up, made good progress, and reported no further episodes. The follow-up time varied from 7 months to 5 years and 3 months. As of October 2021, their ages ranged from 2 to 6 years and 9 months. During follow-up, we surveyed the routine laboratory tests including plasma amino acids and acylcarnitines, urine organic acids and other biochemical investigations. All results were within the normal range. We also summarized patients’ physical growth and developmental quotients (DQ) at the latest follow up, and found normal outcomes in all patients. Both height and weight were between mean±1SD. For the four individuals older than 4 years (Patients 1, 2, 6, and 7), all performed well in school. Additionally, DQ tests revealed that all patients examined had age-appropriate intelligence. Detailed results are shown in Table 1.

### Case Review and Exploration of the Correlation Between Genotype and Clinical Features and Disease Severity

A review of previous literature has revealed that from October 1997 to January 2021, 59 patients, carrying 54 variants of the *HMGCS2* gene, have been reported. Among them, only 11 individuals, from 10 families, were of Chinese descent. Including the 10 cases in our research, 27 mutations have been identified in the 21 Chinese patients from 20 families, including 15 (15/27, 55.6%) missense mutations, 5 (5/27, 18.5%) frame-shift mutations, 4 (4/27, 14.8%) nonsense mutations and 3 (3/27, 11.1%) splicing mutations. The frequency of the *HMGCS2* variants in the 20 unrelated Chinese patients is shown in Table 3. The recurrent *HMGCS2* mutations in the Chinese population were c.1201G > T/p.E401* (detected in 7 individuals from 6 unrelated families, mutated allele frequency 6/40, 15.0%), c.559+1G > T, c.1187+1G > C and c.1502G > A/p.R501Q (respectively detected in 3 mutated alleles, 3/40, 7.5%), c.220G > A/p.E74K (detected in 2 unrelated individuals, mutated allele frequency 2/40, 5.0%).

**TABLE 3:** The frequency of *HMGCS2* variations in 20 unrelated Chinese patients (total number of mutated alleles = 40).

Variation	Region	Nucleotide change	Amino-acid change	Variation type	Variation frequency	Variation	Region	Nucleotide change	Amino-acid change	Variation type	Variation frequency
1	Exon 7	c.1201G>T	p.E401*	Nonsense	6/40 (15.0%)	15	Exon 3	c.616C>T	p.R206C	Missense	1/40 (2.5%)
2	Intron 2	c.559+1G>T	—	Splicing	3/40 (7.5%)	16	Exon 3	c.648G>T	p.M216I	Missense	1/40 (2.5%)
3	Intron 6	c.1187+1G>C	—	Splicing	3/40 (7.5%)	17	Exon 4	c.717T>G	p.Y239*	Nonsense	1/40 (2.5%)
4	Exon 9	c.1502G>A	p.R501Q	Missense	3/40 (7.5%)	18	Exon 4	c.719A>C	p.D240A	Missense	1/40 (2.5%)
5	Exon 2	c.220G>A	p.E74K	Missense	2/40 (5.0%)	19	Exon 4	c.758T>C	p.V253A	Missense	1/40 (2.5%)
6	Exon 2	c.520T>C	p.F174L	Missense	2/40 (5.0%)	20	Exon 4	c.788delT	p.L263Cfs*36	Frame-shift	1/40 (2.5%)
7	Exon 8	c.1347_1351del	p.A450Pfs*7	Frame-shift	1/40 (2.5%)	21	Exon 4	c.821G>A	p.R274H	Missense	1/40 (2.5%)
8	Exon 1	c.39dupA	p.L14Tfs*59	Frame-shift	1/40 (2.5%)	22	Intron 5	c.1017-2A>G	—	Splicing	1/40 (2.5%)
9	Exon 1	c.100C>T	p.Q34*	Nonsense	1/40 (2.5%)	23	Exon 6	c.1175C>T	p.S392L	Missense	1/40 (2.5%)
10	Exon 2	c.160G>A	p.V54M	Missense	1/40 (2.5%)	24	Exon 7	c.1259T>C	p.F420S	Missense	1/40 (2.5%)
11	Exon 2	c.407A>G	p.D136G	Missense	1/40 (2.5%)	25	Exon 7	c.1279C>T	p.Q427*	Nonsense	1/40 (2.5%)
12	Exon 2	c.422T>A	p.V141D	Missense	1/40 (2.5%)	26	Exon 8	c.1394delA	p.N465Tfs*10	Frame-shift	1/40 (2.5%)
13	Exon 2	c.476G>T	p.G159V	Missense	1/40 (2.5%)	27	Exon 9	c.1465delA	p.T489Lfs*55	Frame-shift	1/40 (2.5%)
14	Exon 3	c.563G>A	p.R188H	Missense	1/40 (2.5%)	—	—	—	—	—	—

Including our 10 patients and the previously reported 39 patients with relatively complete clinical data, we analysed the clinical and molecular characteristics of 49 patients to explore the correlation between genotype and clinical features and disease severity. Detailed clinical data is presented in Supplementary Table S1, and the statistical analysis results are shown in Table 4. 16 patients carried biallelic truncating mutations (either nonsense, frame-shift or splicing mutation) (Group A). During acute metabolic decompensation, 4 of them (25%) died, and 7 (43.7%) had severe phenotypes. 13 patients carried monoallelic truncating mutations (Group B). Among them, one patient (7.7%) died, and 10 (76.9%) had severe phenotypes. For the 20 patients carrying biallelic non-truncating mutations (Group C), only one (5%) died, and 7 (35%) showed severe phenotypes, while 12 (60%) had mild symptoms. From another perspective, within the deceased group 66.7% (4/6) had 2 truncating mutations; within the severe group 70.9% (17/24) had at least 1 truncating mutation; and within the mild group 63.2% (12/19) had non truncating mutations. We analysed the correlation between phenotypic groups and genotypic groups with a Cochran-Mantel-Haenszel test. The results indicated that disease severity was correlated with how many truncating variations the patients carried (*p* < 0.05).

**TABLE 4:** Analysis of the correlation between severity of disease and number of truncating variations patients carried.

Group	Deceased (*n*, %)	Severe (*n*, %)	Mild (*n*, %)
A group (*n* = 16)	4, 25%	7, 43.7%	5, 31.25%
B group (*n* = 13)	1, 7.7%	10, 76.9%	2, 15.4%
C group (*n* = 20)	1, 5%	7, 35%	12, 60%
*p*-value	0.0255		

## Discussion

Mitochondrial HMGCS2D is a life-threatening, but treatable, inherited metabolic disorder caused by a defect in the critical enzyme that regulates ketone body formation (Aledo et al., 2006). Therefore, patients with HMGCS2D are prone to episodic metabolic decompensation triggered by fasting or stressful conditions that require fatty acid decomposition for the provision of energy. In the present study, we reported 10 new unrelated Chinese patients with HMGCS2D. All patients had suffered their first metabolic crisis before their first birthday. Prior to metabolic crisis, there had been prodromic infection of either the digestive tract or the respiratory tract in all cases. The most common clinical manifestations before admission were poor intake, fever, cough and vomiting, which progressed rapidly to life-threatening dyspnea and coma. One third of patients had seizures, although each had only one throughout the disease course. Nonetheless, this still suggests there may have been an insufficient energy supply to the brain which may have harmed the nervous system, or even caused sequelae. All of our patients presented with severe metabolic acidosis with significantly increased anion gap, hepatomegaly, increased aminotransferase, and abnormal coagulation function, but normal lactate. Although their primary clinical characteristics were similar to those previously reported, there were several points of concern.

All of our patients, with the exception of Patient 3, had hypoglycemia. According to previously reported data, 3 patients had no hypoglycemia during metabolic decompensation (Lee et al., 2019; Conlon et al., 2020; Wang et al., 2020a). The specific mechanism for this is unknown, and it may be related to compensatory glycogen decomposition and gluconeogenesis. However, the evidence suggested that hypoketotic hypoglycemia was important, but not indispensable, to HMGCS2D diagnosis. Similar to the patients observed by other researchers (Fukao et al., 2014; Conboy et al., 2018), urinary ketones in all of our patients were weakly positive (i.e., not negative). This may have interfered with the initial HMGCS2D diagnosis. Moreover, the observed ketones may have been due to the moderate amounts of ketone bodies produced by leucine catabolism pathway. In addition, in our study, four patients developed hypocalcemia during acute decompensation. Previously, this had only been reported in two patients (Wolf et al., 2003; Kilic et al., 2020). The specific pathogenesis of hypocalcemia remains unclear. We speculate the activation of coagulation and the fibrinolytic system, cell membrane damage caused by severe acidosis and tissue energy deficiency, Vitamin D deficiency and insufficient calcium intake in the acute stage could each be factors. Three patients had transient hyperuricemia, similar to the recent Turkish cases (Kilic et al., 2020). Many organic acids can stimulate the transporter, increasing the reabsorption of uric acid, which may account for the hyperuricemia (George and Minter, 2021). Moreover, Patient 3 had a concomitant disease, congenital laryngeal web, which had been diagnosed when she was 10-months-old based on clinical symptoms including recurrent cough, asthma, laryngeal stridor and hoarseness, and the results of electronic bronchoscopy. Then she accepted interventional therapy, with exceptional results. To our knowledge, this is the first time this has been reported in patients with HMGCS2D.

Detection of metabolic markers are crucial to diagnosing most inherited metabolic diseases. However, as far as we know, there are no specific biochemical markers in HMGCS2D. All patients in this study showed elevated dicarboxylic acid in their urine during decompensation. It has been reported that this can provide an indication, but cannot differentiate HMGCS2D from other fatty acid metabolic disorders (Kilic et al., 2020; Wang et al., 2020b). It has been reported that increased urine 4-hydroxy-6-methyl-2-pyrone (4-HMP) can be a specific biochemical hallmark indicator which can distinguish HMGCS2D from fatty acid oxidation defects (Pitt et al., 2015; Wang et al., 2020a). Unfortunately, in our study, only 3 patients received urine 4-HMP tests. Among them, 2 had elevated urinary 4-HMP and the other patient’s urinary 4-HMP was normal. These results indicated that using 4-HMP as a positive prediction needs further study through more patients. We also detected the blood acylcarnitine profile in all individuals. As previously reported in the literature, blood acetylcarnitine (C2) can be either normal or increased during decompensation (Aledo et al., 2006; Ramos et al., 2013; Liu et al., 2019). In our study, the blood C2 level in 8 of the patients increased, while the free carnitine (C0) level in 7 of the patients decreased. This led to elevated C2/C0 ratios in all cases. These data suggest that for clinically suspected patients, the high ratio of C2 to C0 may have been more specific and informative than the solitary high C2 or low C0 levels. Consequently, we considered that simultaneous detection of blood acylcarnitine profile and urinary organic acids was essential for HMGCS2D diagnosis. An elevated C2/C0 ratio combined with plenty of dicarboxylic acids in urine during an episode of acute hypoglycemia and metabolic acidosis may be an additional biochemical signature of HMGCS2D. This observation needs further research to identify whether there is sufficient specificity for clinical utilization.

As mentioned above, the lack of specificity in clinical and biochemical characteristics, as well as any abnormalities, may turn normal during intermissions. This complicates HMGCS2D diagnosis. On the other hand, detecting enzyme activity is invasive and complex, and the results are unstable. Therefore, at present, molecular genetic analysis of the *HMGCS2* gene still consists of the recommended diagnostic method (Zschocke et al., 2002; Ramos et al., 2013). In recent years, more and more patients have been confirmed as carrying biallelic pathogenic variants on *HMGCS2* gene by genetic testing. The *HMGCS2* gene is located on chromosome 1p13-p12, with a length of 20.9 kb. It consists of 10 exons, encoding 508 amino acids. Since the first description of the disease in 1997 (Thompson et al., 1997), over 40 variants have been identified. Prior reports have shown that these mutations have an irregular distribution and involve all exons (Wang Q. et al., 2020). Most of them are sporadic, specific to families, and have ethnic and regional differences. The c.634A > G/p.G212R has been identified in 3 families, respectively from Poland (Conlon et al., 2020), Britain (Pitt et al., 2015) and Germany (Zschocke et al., 2002). This implies that it was a common HMGCS2D mutation in Europe. The large deletion on exon 1, denoted as c.1-?_104+?del, has only been identified in patients of Mediterranean descent (Pitt et al., 2015), while c.725-2A > C has only been reported in two families from Turkey (Kilic et al., 2020). No relevant research on phenotype-genotype correlations has been reported to date.

Our finding is unlike similar studies conducted on other populations. In our cohort, the recurrent variants were c.1201G > T/p.E401*, c.559+1G > T and c.220G > A/p.E74K. If combined with the 11 patients reported previously (Thompson et al., 1997; Ma and Yu, 2018; Liu et al., 2019; Wang et al., 2019; Wang et al., 2020a; Wang et al., 2020b; Yang et al., 2020), the most frequent mutation was c.1201G > T/p.E401*. This may result in a premature termination codon at amino acid residue 401 located in exon 7 of *HMGCS2*. This leads to a truncated protein, and causes the loss of gene function. We detected this mutation as compound heterozygous in 7 Chinese patients from 6 families, while no patient in any other population has been a carrier of c.1201G > T/p.E401* in prior studies. All of these observations suggested that c.1201G > T/p.E401* may be a hot-spot mutation in Chinese HMGCS2D patients. The second most common mutations were c.559+1G > T, c.1187+1G > C and c.1502G > A/p.R501Q, all accounting for 3/42 mutated alleles, repectively. All had been reported in previous Chinese HMGCS2D patients, but not in other populations. c.559+1G > T was reported to be the second described splicing mutation of the *HMGCS2* gene, and minigene assay results revealed that it may influence protein structure and function (Wang et al., 2020b). c.1187+1G > C is the classical site splicing mutation, which may cause abnormal splicing resulting in loss of protein function. We detected the missense mutation c.1502G > A/p.R501Q in Patient 6 in the compound heterozygous state, and a previous study has reported a Chinese case in the homozygous state (Ma and Yu, 2018). Previous reports have also suggested that arginine at position 501 is critical to enzymatic function (Bagheri-Fam et al., 2020). Moreover, variation in amino acids in the same position, c.1502G > C/p.R501P, has also been found in two Thai patients (Rojnueangnit et al., 2020). All of these factors imply that it was a causative variant.

Furthermore, our study also identified 10 novel mutations of *HMGCS2*. The c.1017-2A > G was the fifth splicing mutation of *HMGCS2*. It occurred at a classical splicing site and may lead to abnormal formation of *HMGCS2* protein, demonstrating its pathogenicity. c.717T > G/p.Y239*, c.39dupA/p.L14Tfs*59, c.788delT/p.L263Cfs*36 and c.1394delA/p.N465Tfs*10 all led to a premature termination codon respectively in exons 4, 1, 4, and 8. This led to encoded truncated peptides, thereby resulting in an enzyme defect. The 5 missense mutations each occurred in highly conserved regions. They were predicted to be damaged by Mutation taster, SIFT, and Polyphen 2. The computational evaluation using Swiss-model software showed that they may affect the 3D structure of HMGCS2 protein. Among them, c.220G > A/p.E74K was detected in two unrelated patients. We speculated that it is a common mutation in Chinese patients.

In order to explore the correlation between genotype and clinical features and disease severity, which had remained unknown according to previous studies, we analysed the clinical and molecular characteristics of 49 patients. This included our 10 patients, as well as the previously reported 39 patients with relatively complete clinical data (as shown in Supplementary Table S1). In Table 4, Group A (patients carrying biallelic truncating mutations) had the highest mortality (25%), while Group C (patients carrying biallelic non-truncating mutations) had the highest percentage of mild cases (60%). Group B (carrying monoallelic truncating mutations) had the highest percentage of severe cases (76.9%). Statistical analysis showed a correlation between disease severity and how many truncating variations patients carried. This suggests that patients with biallelic truncation mutation are likely to have more severe phenotypes. However, there was no convincing evidence indicating that individuals with biallelic missense mutation would have milder or later presentation. On the other hand, due to the limited number of patients, the frequency of compound heterozygotes, and the lack of enzymatic studies, it is difficult to assess the precise relationship between genotype and phenotype. Furthermore, inter-allelic complementation complicated the predictions of potential genotype-phenotype correlations. Therefore, more cases are needed for further exploration.

With regard to the prognosis, most of our patients had full recovery after symptomatic and supportive treatment, and maintained normal growth and development, without further episodes during follow-up. According to the literature, only one case had neurological sequelae (Conboy et al., 2018). However, there still were 6 patients who died of hypoglycemic crisis and serious metabolic acidosis during acute metabolic decompensation (Liu et al., 2019; Kilic et al., 2020; Wang et al., 2020a; Yang et al., 2020). These results suggest that HMGCS2D is a fatal, but treatable, hereditary metabolic disease. If we can diagnose it before onset, and then prevent those trigger factors like long-term fasting, and administrate oral glucose when patients have poor food intake, we may be able to prevent metabolic crises. However, patients cannot be diagnosed before disease onset through the current newborn screening program. We believe that the combination of rapid and accurate next-generation sequencing technology and biochemical screening may improve newborn HMGCS2D screening efficiency in the future (Luo et al., 2020).

## Conclusion

In summary, mitochondrial HMGCS2 deficiency is a rare ketone synthesis disorder. We have described clinical symptoms, biochemical features, clinical outcomes, and molecular analysis of 10 new Chinese patients. We have also expanded the *HMGCS2* mutational spectrum with 10 novel variants. The variation c.1201G > T/p.E401* is the most common, and may be a hot-spot mutation of the *HMGCS2* gene in Chinese patients. Through a literature review, we found that patients with biallelic truncation mutation appeared to show a more severe clinical condition.

## Data Availability

The data that support the findings of this study are submitted to ClinVar through ClinVar Submission Portal and available from the corresponding author upon reasonable request. Accession link to HMGCS2 cDNA sequence NM_005518 https://www.ncbi.nlm.nih.gov/nuccore/NM_005518.4. ClinVar accession link of new variants of HMGCS2 gene identified in this study https://www.ncbi.nlm.nih.gov/clinvar/?term= SUB10769664.
